# The Role of Cingulate Cortex in Vicarious Pain

**DOI:** 10.1155/2015/719615

**Published:** 2015-02-26

**Authors:** Esther H. Yesudas, Tatia M. C. Lee

**Affiliations:** ^1^Laboratory of Neuropsychology, The University of Hong Kong, Room 656, The Jockey Club Tower, Pokfulam Road, Hong Kong; ^2^Laboratory of Social Cognitive Affective Neuroscience, The University of Hong Kong, Room 656, The Jockey Club Tower, Pokfulam Road, Hong Kong; ^3^The State Key Laboratory of Brain and Cognitive Sciences, The University of Hong Kong, Room 656, The Jockey Club Tower, Pokfulam Road, Hong Kong; ^4^Institute of Clinical Neuropsychology, The University of Hong Kong, Room 656, The Jockey Club Tower, Pokfulam Road, Hong Kong

## Abstract

Vicarious pain is defined as the observation of individuals in pain. There is growing neuroimaging evidence suggesting that the cingulate cortex plays a significant role in self-experienced pain processing. Yet, very few studies have directly tested the distinct functions of the cingulate cortex for vicarious pain. In this review, one EEG and eighteen neuroimaging studies reporting cingulate cortex activity during pain observation were discussed. The data indicate that there is overlapping neural activity in the cingulate cortex during self- and vicarious pain. Such activity may contribute to shared neural pain representations that permit inference of the affective state of individuals in pain, facilitating empathy. However, the exact location of neuronal populations in which activity overlaps or differs for self- and observed pain processing requires further confirmation. This review also discusses evidence suggesting differential functions of the cingulate cortex in cognitive, affective, and motor processing during empathy induction. While affective processing in the cingulate cortex during pain observation has been explored relatively more often, its attention and motor roles remain underresearched. Shedding light on the neural correlates of vicarious pain and corresponding empathy in healthy populations can provide neurobiological markers and intervention targets for empathic deficits found in various clinical disorders.

## 1. Introduction

Empathic understanding of the affective state of others in pain may involve cognitive, affective, and motor processes facilitated by brain areas involved in both the direct experience of pain and its observation [1, 2]. Whereas the function of many of such neural correlates is not yet well understood, it has been consistently proposed that the cingulate cortex contains neuronal populations that underpin self-pain and other-pain processing. Indeed, the association between cingulate cortex activity and self-experienced pain has been well established [3, 4]. Likewise, activity in this region has been reported when individuals perceive pain in others [1]. However, the functions of the cingulate cortex are not all pain-specific and a part of its activation in pain experiences may subserve its roles in affect, attention, and motor preparation [5–7]. While this has been extensively researched for self-pain, very few studies have directly tested the distinct functions of the cingulate cortex for vicarious pain. Consequently, it remains unclear not only what the exact role of this region is during pain observation, but also whether the specific activations are shared or distinct for self-pain and witnessed pain. This paper reviews neuroimaging literature that reveals cingulate cortex activity during the perception of pain in others with the aim of highlighting current research gaps to shed light on the possible directions of future research. The review will begin by introducing a model that explains the neural mechanisms underlying empathic understanding. Thereafter, it will present studies which investigated the cingulate cortex in its affective, attentional, and motor functions during pain observation. Implications, limitations, and future outlook will be provided.

## 2. Vicarious Pain

Vicarious pain is defined as the observation of another individual in pain [8]. This process can elicit empathy which is characterized by an understanding of their emotional state and induction of corresponding states in the self that remain conceptually separate from personal distress [9, 10]. Thus, empathizing with pain observed in another individuals can have comparable effects as self-pain which is experienced on the own body [11, 12]. Reflecting this similarity, neuroimaging literature indicates that empathy may arise from distinct and shared neural representations that underlie self-pain and pain in others [13, 14]. The perception-action model (PAM) of empathy integrates both types of representations [11]. It relies on the mirror-neuron system which is characterized by the so-called mirror neurons that show activation both when an action is actively performed as well as when it is passively observed [15]. Accordingly, when passively witnessing pain, mirror neurons that correspond to the muscles involved in active self-pain are activated. In consequence, a neural network that contains learned sensory and affective information for self-pain that can be used to evaluate the suffering of others is stimulated. This, in turn, facilitates empathy [13]. Research provides evidence for mirror neuron activity in empathy to vicarious pain that is distinct from self-experienced pain. In particular, the inferior frontal gyrus (IFG) and inferior parietal lobule (IPL) as core regions of the human mirror-neuron system respond to observed pain while remaining inactive during self-pain [1, 8, 14–17].

### 2.1. Cingulate Cortex and Pain Perception

The cingulate cortex, on the other hand, may play a role in shared mirroring and representations. It is part of the neural pain matrix that activates during self-experienced pain which further includes the primary (SI) and secondary somatosensory cortices (SII), thalamus, prefrontal cortex (PFC), and insula (INS), while the SI, SII, and thalamus subserve sensory processing of pain stimuli, reflected in pain intensity ratings, the PFC, INS, and cingulate cortex, are involved in cognitive-affective evaluation, reflected in pain unpleasantness ratings, and top-down control [3, 4, 18–20]. In line with the PAM, similar cingulate cortex activity is revealed for self-pain and others pain. However, it is debated whether this activity occurs in identical neural populations or in adjacent areas in the cingulate cortex [2, 21, 22]. Identical populations would provide evidence for shared neural substrates of self- and vicarious pain processing. In contrast, adjacent activation may reflect other cingulate cortex functions that are involved in the representation of affective or sensory experiences in general. Both shared and distinct neural activity may facilitate empathy to the observed pain by activating the existing associations or contributing cognitive factors to evaluate the state of others. Research that explores cingulate activity during vicarious pain has reported both shared and distinct activations. However, clarification is needed to determine which functions are specifically subserved by the detected neural activity.

Using all available ProQuest databases, a search with the keywords* pain* AND* empathy* AND (*cingulate cortex*) came back with 1,194 results. From the search results, titles and abstracts were assessed according to these inclusion and exclusion criteria and a smaller sample was extracted for in-depth evaluation. During the initial screening of titles, all nonimaging studies, animal research, and studies testing interventions were excluded. During further evaluation stages, only studies directly investigating neural correlates underlying vicarious pain in healthy populations and reporting on cingulate activity during pain observation were included in this review. In the final screening, all papers investigating neural modulation of empathy through individual differences or clinical conditions were excluded. The final sample for this review consists of eighteen neuroimaging studies and one EEG study. Among the sample, previous research findings indicating that different cingulate cortex areas are associated with distinguished functions were reflected (for reviews, see [1, 23, 24]). While the ventral regions of the cingulate cortex (anterior cingulate cortex; ACC) tend to be involved in emotional processing and learning [5, 25], the dorsal regions (anterior midcingulate cortex: aMCC) show greater activation during motor tasks, attention, and response selection [23]. Both areas are implicated in self- and vicarious pain. During self-experienced pain, ACC activity correlates with affective pain factors while aMCC activity is linked to both affective and sensory processing [18]. Furthermore, the aMCC is associated with salience detection, which signals the need for attention to threat stimuli [26], and motor planning in response to pain stimulation via own motor regions and connections to the motor cortices [25]. ACC and aMCC activities are also reported for vicarious pain perception [1]. Corradi-Dell'acqua et al. [2] propose that some of these activities overlap with responses during self-pain while others are distinct. The roles of these regions in self-pain may be comparable to vicarious pain suggesting that empathy arises from the processing of various stimulus dimensions [2, 27]. The three main functions ascribed to the ACC and MCC are affect, attention, and motor preparation. While affective processing is more frequently explored, attention and response selection during vicarious pain are underresearched with very few studies utilizing paradigms that specifically target these two areas. For an overview of all studies including stimuli, instructions, and findings, please refer to Table 1.

### 2.2. Affective Processing in Vicarious and Direct Pain 

The ACC and MCC are involved in affective processing of self-experienced [3, 4] and vicarious pain [1]. Vicarious pain studies suggest that affective neural activity represents the unpleasantness associated with observed pain and facilitates inference of the affective state of the suffering individual. In line with this, studies report increased activity in the subgenual and rostral ACC (sgACC; rACC) and aMCC during pain observation in limbs and facial expressions [21, 28, 29]. These areas are relevant for the emotional processing of aversive stimuli. Facial expressions and cues signaling pain in others have been attributed to affective processing as they require individuals to decode emotional information often without knowledge about the noxious stimulus. The sgACC, rACC, and aMCC are implicated in such decoding through shared affective mirroring in which similar neurons fire when observing facial affect as when experiencing this affect [8, 21, 28, 29]. Indeed, ACC activations were found to be partially overlapping and partially anterior to activations in self-pain supporting both shared pain affect representations and distinct contributions [21, 22]. Furthermore, rACC activity increased with higher emotional attachment to the person in pain even when their affect was not viewed, suggesting pain empathy may be facilitated by interpersonal relationships [30]. Similar to self-pain reports [31, 32], sgACC and rACC activation correlated with increased pain evaluations [21] and empathic distress [30]. Nonetheless, despite the correlations between perceived facial pain expressions and neural activity, the functions of this activity cannot be confidently determined as neuroimaging studies do not evaluate pain unpleasantness ratings as specific behavioural measurements of affective pain responses [28, 29]. Thus, it remains unclear whether the cingulate cortex subserves affective processing or fulfils other roles that contribute to empathy induction.

In contrast to facial expressions, the perception of limbs in pain is assumed to evoke both sensory and affective pain evaluations as the noxious stimuli are known. Currently, two studies using such stimuli correlated unpleasantness ratings have found positive correlations to ACC activity. This observation supports the involvement in affective processing of vicarious pain [22, 33]. However, a similar role for the aMCC is not confirmed. Jackson et al. [10] reveal that while the ACC is active only when the observed pain is imagined from a first-person perspective, aMCC activation occurs during both first- and third-person perspectives. Likewise, Lamm et al. [33] report that the aMCC was active both when participants focused on the sensory and affective pain components, while the ACC was linked only to affective focus. Therefore, it is likely that the functions of the aMCC are not specific to affective processing; furthermore, its activity may also not be pain-specific [34]. Indeed, lesion studies indicate that these regions are not essential for empathy induction [35]. Moreover, although the ACC and aMCC are associated with higher empathic traits, indicating that greater neural processing may facilitate empathy, exact locations for shared affective pain representations remain to be confirmed [2, 8, 29]. Consequently, while affective pain processing may facilitate empathy by stimulating shared associations for self-pain and others pain which permit inference of emotional states in others [21, 30, 36], the ACC and aMCC may have functions during pain observation that go beyond shared pain representations and are neurally shared or distinct from self-pain [37].

The ACC and aMCC play significant roles in emotional regulation of own responses. Gu and Han [38] noted increased functional connectivity between the ACC and inferior frontal cortex during painful images and corresponding decreased emotional reactivity, suggesting top-down regulation of ACC activity through the frontal regions, similar to self-pain processing. Furthermore, Lamm et al. [39] reported increased aMCC activity and functional connectivities between aMCC and both the periaqueductal gray (PAG) and dorsomedial prefrontal cortex (dmPFC) when participants observed hands being painfully pricked by a needle to which hand models were said to react with no pain. It was suggested that the affective components of aversive pain incidents are processed in the aMCC. However, when it is understood that the observed scenario is aversive only to the self, but not to the involved individual, downregulatory feedback is sent to the aMCC from frontal structures, such as IFC and dmPFC, and pain-regulatory regions, such as PAG. This allows the for the incorporation of cognitive information and contributes to a more accurate evaluation of the affective state of the other, leading to more context-appropriate empathy [39]. When practiced regularly, emotional regulation occurring at an early stage may help free up cognitive resources for response formation [40]. As with brain activations, increased functional connectivities also resemble those during self-experienced pain [41]. Nonetheless, it remains unclear whether the specific pathways of functional connectivity overlap or are adjacent during both pain experiences [2, 36]. Moreover, the relationship between these connectivities requires further direct testing to substantiate prior findings before firm conclusions can be drawn. Comparable to self-pain, emotional regulation during pain observation seems to involve the cingulate cortex and frontal regions and may contribute to more accurate empathic understanding.

### 2.3. Attention Processing in Vicarious and Direct Pain 

In vicarious pain studies, the role of the aMCC in attention and cognitive processing is often neglected [35]. During self-pain, the aMCC is implicated in automatic attention toward salient threat cues and voluntary attention focus to chosen stimuli such as cognitive tasks [42–45]. Higher task demand is associated with greater aMCC activation, reflecting the increased need for attention [46]. Although substrates for self-pain processing are posterior to those for attention, directing attention to cognitive tasks has been consistently found to decrease pain reports and corresponding brain activation, suggesting that distinct neuronal populations in the same brain area can impact each other [7, 46, 47]. In line with this thought, the limited capacity model (LCM) proposes that as stimuli compete for finite resources, increasing focus on one stimulus withdraws these resources from other stimuli and thus attenuates them [48]. Up to date, only three neuropsychological studies have addressed attention in vicarious pain. In an EEG study, Fan and Han [49] confirmed that the identification of observed pain compared to no-pain and empathic evaluation occurs earlier when attention is focused on rather than away from the pain. Moreover, in an fMRI study, Gu and Han [38] presented participants with images of hands in painful or neutral scenarios and asked them to either attend to pain cues or count hands. As in previous studies, aMCC activation was found when attending to painful rather than neutral images. However, when comparing counting hands in painful to neutral images, aMCC activation became nonsignificant. This indicates both that attending away from pain cues inhibits automatic pain processing as well as that aMCC responses to painful images can underlie attention rather than being pain-specific. Subsequent studies support this, demonstrating that when tasks require similar attention levels, aMCC activation occurs during the observation of both painful and neutral images without significant increase in response to vicarious pain. This further highlights the role of the aMCC in attention rather than affective pain processing [35]. In line with LCM, aMCC activity decrease may reflect the withdrawal of neural resources from pain-related processing in favour of attention. The elimination of pain-related activity in these studies indicates that pain empathy correlates are more susceptible to attention modulation than self-pain, possibly because they are less salient. The findings advocate attention as a function subserved by the aMCC during vicarious pain similar to self-pain and highlight the research gap in vicarious pain. It is possible that those attention correlates contribute to shared neural representations of self-pain and others pain or add distinct processing to facilitate empathy [38]. The distinct aMCC areas of pain and attention processing during self-pain have not yet been replicated for vicarious pain, although it has been suggested that like self-pain, other-pain processing regions are posterior to attention processing [2, 27]. Whether the aMCC may have a similar role in attention during vicarious as that during self-pain awaits verification in future research. Furthermore, studies to examine whether the neural correlates of vicarious pain in affective processing regions are shared or distinct from self-pain are worthwhile.

The involvement of the aMCC in attention during vicarious pain is further supported by neuroimaging findings. In self-pain, Seminowicz and Davis [50] propose that pain and attention processing rely on functional connectivities between aMCC and prefrontal regions. In vicarious pain, similar connectivities have been proposed [39]. Furthermore, previous research findings have indicated that asking participants to maintain attention toward sensory aspects of vicarious pain was associated with activation in somatosensory regions compared to focus on affective pain aspects which activates affective regions [8, 33, 38, 51–53], and there are also findings showing increased functional connectivity during sensory focus between the aMCC and the motor cortices which in turn display higher connectivity with the somatosensory cortices [27, 54, 55]. Thus, there is a neural effect of attention focus in vicarious pain that impacts communication to relevant brain areas. Furthermore, salience detection is reflected in increased neural synchronization between aMCC and the superior temporal sulcus (STS). The STS is associated with the affective evaluation of salient social cues and, as such, the aMCC may direct attention to the implications of vicarious noxious stimulus and send information to the STS for further evaluation [54]. Finally, increased connectivities between visual attention areas and the aMCC may support attention maintenance on the threat cues and potential pain anticipation [27, 35]. Given these findings, it is plausible that the aMCC has a similar role in attention for vicarious and self-pain. However, the exact substrates and connectivities still need to be established.

### 2.4. Motor Preparation in Vicarious and Direct Pain 

The role of the aMCC in preparatory motor activity and response selection has been well-established for self-pain, but it has not yet been directly tested for vicarious pain. The aMCC includes two motor areas, the caudal cingulate and the rostral cingulate zone which project to the motor cortices [25, 56]. In self-pain, the aMCC responds to the presence of conflicting response choices, such as pain avoidance or tolerance, with increased activity and functional connectivity to the motor cortices where a preparatory motor response is formed [57, 58]. Accordingly, this brain region is involved in coordinating responses to noxious stimuli in line with motivational urges, for example, withdrawal from pain. It is theorized that it fulfils similar functions in vicarious pain. Morrison et al. [36] subjected participants to animations of items striking or missing hands. These items were either noxious or neutral and participants indicated via button press whether the items struck or missed the presented hands. Increased reaction times were found only for noxious implements in the strike condition. This Stroop Effect was proposed to result from the need to inhibit motor preparation of automatic withdrawal urges from the observed pain in order to press the correct button, yet further investigation is required to rule out alternative explanations. It is also possible that if pain affect, attention, and motor preparation drawn from the same brain region, the Stroop Effect could be a consequence of resource sharing [38, 48]. Nonetheless, increased activity in the aMCC, specifically in the caudal cingulate zone, was reported when the noxious implements were shown to strike the hand and participants had to give a motor responses compared to passive pain observation. This suggests that, similar to self-pain, motor preparation may occur in the motor zones of the aMCC during vicarious pain. Morrison et al. [36] concluded that the neuronal populations in the aMCC that activate to pain and motor processing lie adjacent to another and interact during vicarious pain. It is possible that the neurons in the aMCC are involved in motor mirroring as proposed by the PAM, suggesting that neurons underlying muscles involved in self-pain are also responsive during observed pain, stimulating shared associations to prepare for motor movement. However, further research is required to assess this. The involvement of the aMCC in motor preparation is further substantiated through its increased connectivity to the motor cortices during pain observation [36, 55, 59] as well as fMRI and TMS studies that support the involvement of motor cortices in vicarious pain [28, 33, 52]. In particular, TMS studies are robust as they give causational evidence. Consequently, the motor zones of the aMCC may be a further platform for shared neural representation between self-pain and others pain or distinct contributions to vicarious perception. Although many vicarious pain studies explain aMCC activation in terms of motor preparation, no studies have explored this directly so far [27, 29].

## 3. Translational Values and Future Research

Vicarious pain research reports comparable cingulate cortex activations for self- and other-pain. While these are suggested to subserve affective pain processing, attention, and motor preparation, only few studies have tested these functions directly for vicarious pain [21, 22, 28, 34, 38, 39]. Moreover, there is evidence for neuronal populations in this brain region that create shared neural representations for self- and other-pain that may stimulate associations used to evaluate the emotional states of observed individuals in pain. However, exact locations and pathways of overlapping neural activity have not yet been established. Furthermore, there may be adjacent neuronal populations that make distinct contributions to empathy toward vicarious pain. Greater insight into the neural underpinnings of empathy is essential for the detection of empathic deficits that are pertinent to various disorders, including schizophrenia [60, 61] and motor neuron disease [62]. Blunted empathy responses are associated with dysfunctional pain, affect, attention, and motor processing as well as altered cingulate cortex activity or structure correspondingly [62–65]. For example, individuals diagnosed with attention-hyperactivity deficit disorder (ADHD) show dysfunctional aMCC activation during cognitive tasks as well as impaired emotion recognition in others [63]. Nonetheless, the relationship between cingulate impairments, empathic deficits, and pain processing is not straightforward. While individuals with congenital insensitivity to pain report impaired sensory pain perception, fMRI studies reveal aMCC activity and empathy responses that reflect those of healthy controls. This strongly suggests that aMCC function extends beyond shared neural pain representations in empathy induction [66]. Likewise, paradoxically, decreased grey matter in the right ACC in alexithymia, which is highly associated with clinical depression, has been found to correlate with blunted empathy toward other-pain but increased pain sensitivity during self-pain [65, 67, 68]. Such dissociation is likely to be explained through distinct neural correlates of empathy rather than shared representations. Clinical findings highlight the involvement of the cingulate cortex in self-pain, vicarious pain, and empathy. Defining its role clearly is crucial for identifying neurobiological markers of empathic deficits and can provide intervention targets.

The available literature for neural correlates of vicarious pain is at its beginning. Future research is required to tease apart the cingulate cortex activity during observed pain that is responsible for affect, attention, and motor preparation and differentiate shared and distinct neural representations that evoke empathy. Forthcoming studies may include behavioural measures to determine functions of the relevant brain activity as well as factors impacting empathy or pain perception to explore how the link between empathic affect and behaviour is moderated and reflected in neural activity [29, 51, 69–71]. In a clinical context, few studies exist that investigate neural activity in individuals with empathic deficits compared to healthy controls (e.g., [65]), top-down regulation of empathic responses (e.g., [40]), or the effects of vicarious pain on self-pain perception (e.g., [72]). Further exploration can advance the knowledge on neurobiological indicators of dysfunctional empathy.

Despite the great potential for future research, pinpointing neural correlates of vicarious pain is subject to several challenges. First, empathy research has to differentiate paradigms that entail passive stimuli viewing from those that requiring affective inferences as they are likely to recruit different brain processes. Also, artificiality of the laboratory environment has to be considered when interpreting results as the induction of empathy in the real world is likely to be a complex process [73]. Second, neuroimaging tools preclude concurrent measurement of activity location and time sequence as they are high on either spatial or temporal resolution and individual brain differences may further reduce location accuracy. Moreover, they may not be sensitive enough to record weaker activity; hence, other activations cannot be excluded beyond doubt [33, 38, 49]. Likewise, in the direct comparison of self- and vicarious pain neural activity, their stimuli differ on multiple levels, engaging distinct brain areas, and are being of qualitatively different feel [54]. Thus, distinguishing their specific signal changes from those of other factors in the paradigm can be difficult [10, 21, 36]. Nonetheless, it is hopeful that current analysis methods are improving. For example, the multivariate pattern analysis (MPVA) is progressing toward more fine-grained analysis of brain activity and its location [2]. Future research can tackle such existing challenges and increasingly shed light on the involvement of the cingulate cortex in empathy to vicarious pain and inform on shared and distinct neural representations for self- and other-pain.

## 4. Conclusions

This review has shown that there is evidence for overlapping neural activity in the cingulate cortex during self- and vicarious pain. Such activity may contribute to shared neural pain representations that permit inference of the affective state of the individual in pain, facilitating empathy. Nonetheless, future research is required to confirm and establish the exact location of these shared neural representations. Furthermore, neuronal populations that are distinct from self-pain and thus specific to vicarious pain may make unique contributions to the empathic experience. Importantly, the different functions of the cingulate cortex should be kept in mind when exploring vicarious pain correlates. Activity in this brain region may underpin cognitive, affective, and motor processing for both self- and others pain. Future research should address the current lack of research specifically teasing apart these distinguished types of processing for vicarious pain to further define the exact role of differing neural activation in inducing empathy. Findings are of significance to clinical practice in which empathy deficits characterize a wide variety of disorders, such as ADHD, depression, and schizophrenia. Establishing structural and functional cingulate cortex processes during vicarious pain in the healthy population may provide both neurobiological markers for empathic deficits and potential intervention targets.

## Figures and Tables

**Table 1a:** (a) Affective Processing

Author and date	Stimuli	Task	Activation	Findings
*Facial expressions *				

Botvinick et al. (2005) [29]	Videos of faces in pain or no pain and self-pain or no pain.	No rating.	ACC	ACC activation during self- and vicarious pain in facial expressions.

Budell et al. (2010) [21]	Videos of facial expressions of pain.	Rate pain experience on VAS.	sgACC rACC aMCC	ACC and aMCC activation during vicarious pain is more anterior than for previous findings in self-pain. Pain ratings correlated with sgACC activity.

Lamm et al. (2010) [39]	Images of hands deeply penetrated by needle or touched by Q-tip and facial expressions.	Rate pain intensity on VAS while sharing affect.	ACC aMCC	Activation during vicarious painful stimulation with nonpainful object and nonpainful stimulation with painful object. Empathic traits correlate with ACC. Increased functional connectivity in MCC, aMCC, PAG, and aINS during vicarious pain.

Saarela et al. (2007) [28]	Images of faces of chronic pain patients at resting state of chronic pain or during provoked acute pain.	Rate pain intensity and unpleasantness on Likert scale.	ACC	ACC activation during vicarious pain.

Vachon-Presseau et al. (2012) [8]	Images of hands or feet subjected to pain or no pain and facial expressions of pain.	Rate pain experience on VAS.	aMCC	Activation during vicarious pain in limbs and facial expressions. Empathic traits correlate with aMCC activation. Affective-motivations response of vicarious pain is independent of channel of pain.

*Limbs in pain *				

Corradi-Dell'acqua et al. (2011) [2]	Images of hands in pain or no pain.	Rate pain intensity on Likert scale.	MCC	Distinct neural populations involved in affective pain processing, attention, and motor preparation. Overlapping neural populations for self- and vicarious pain.

Jackson et al. (2006) [10]	Images of hands or feet in pain or no pain.	Rate pain level on VAS from first- or third-person position.	ACC aMCC	ACC associated with taking first-person position during vicarious pain. Pain rating correlated with ACC. aMCC associated with both self- perspective and the perspective of others during vicarious pain.

Jackson et al. (2005)	Images of hands or feet in pain or no pain.	Rate pain level on VAS.	ACC aMCC	ACC activation during vicarious pain. No correlation between empathic traits and ACC and self-reported pain sensitivity.

Lamm et al. (2007) [33]	Images of hands deeply penetrated by needles.	Rate sensory or affective qualities of pain on VASs.	sgACC aMCC	sgACC associated with pain unpleasantness ratings. aMCC activated during both sensory pain and affective pain focuses on pain.

Morrison and Downing (2007) [22]	Video of needle deeply penetrating hand or self-experienced needle penetrating hand.	Rate pain unpleasantness.	ACC	ACC activity adjacent and overlapping between self-pain and others pain. ACC associated with pain unpleasantness ratings.

Morrison et al. (2004) [27]	Videos of hands experiencing pin prick or self-experienced pin prick.	Rate pain unpleasantness on Likert scale.	ACC	Activation during vicarious pain and self-pain.

Ochsner et al. (2008) [59]	Videos of individuals subjected to pain or self-experienced heat pain.	No rating.	aMCC	Activation during vicarious and self-pain. Increased functional connectivity between aMCC, aINS, and mPFC during vicarious pain.

Ogino et al. (2007) [34]	Neutral, fear, and pain images. Images of limbs in pain.	Imagine observed pain in own body.	aMCC	aMCC activation during vicarious pain and fear images.

Osborn and Derbyshire (2010) [51]	Images or short clips of limbs or full individuals subjected to pain.	Rate pain intensity on VAS.	rACC	rACC activation during vicarious pain in participants who could feel pain in their own body and those that could not.

Zaki et al. (2007) [54]	Videos of individuals subjected to pain or self-experienced heat pain.	No ratings.	ACC	Increased functional connectivity between ACC, aINS, and dmPFC during vicarious pain. Increased functional connectivity between ACC, STS, PCC, and precuneus during vicarious pain.

*Abstract cues *				

Singer et al. (2004) [30]	Abstract cue that loved one receives electric pain or self-experienced electric pain.	Rate pain unpleasantness.	rACC	rACC correlated with empathy scores.

**Table 1b:** (b) Attention

Author and date	Stimuli	Task	ACC activations	Findings
Gu and Han (2007) [38]	Images or cartoons of hands in pain or no pain.	Attend to pain cues or count hands in image.	aMCC	Greater functional connectivity to left inferior frontal cortex (top-down regulation) during vicarious pain. Pain-related activation in aMCC eliminated when attention was withdrawn from pain.

Gu et al. (2010) [35]	Images of limbs in pain or no pain.	Judge if it is painful or not painful and laterality.	aMCC	No difference in activation between painful and nonpainful stimuli or judgment types. Increased functional connectivity during vicarious pain with visual attention areas and anterior insula.

Fan and Han (2008) [49]	Images or cartoons of hands in pain or no pain.	Attend to pain cues or count hands in image.		Early differentiation between pain and no pain.

**Table 1c:** (c) Motor preparation

Author and date	Stimuli	Task	ACC activations	Findings
Morrison et al. (2006)	Short animations of a noxious or nonnoxious item, striking or not striking a hand.	Report whether hand in animation was struck or not struck by item via button press.	aMCC MCC	Increased aMCC activation during vicarious pain and required motor responses. Link between motor inhibition and MCC activity in caudal cingulate motor zone.
